# HBV re-activation is associated with increased mortality and dysregulation of oxidative and polyamine metabolism in pre-acute-on-chronic liver failure

**DOI:** 10.1186/s12964-026-03145-y

**Published:** 2026-08-24

**Authors:** Mirco Glitscher, Keerthihan Thiyagarajah, Jianyi Wei, Jannik Sonnenberg, Pia Lembeck, Wenyi Gu, Georgios Grammatikos, Lining Guo, Jonel Trebicka, Guohong Deng, Xianbo Wang, Xin Zheng, Yan Huang, Jinjun Chen, Zhongji Meng, Yanhang Gao, Zhiping Qian, Feng Liu, Xiaobo Lu, Yu Shi, Yubao Zheng, Yingli He, Hai Li, Eberhard Hildt, Kai-Henrik Peiffer

**Affiliations:** 1 Medical Clinic B, University Hospital Münster, Albert- Schweitzer Campus 1, D48149 Münster, Münster, Germany; 2 Research Group 2, Paul-Ehrlich-Institut, Langen, Germany; 3 Division Gastroenterology, University Hospital Frankfurt, Frankfurt am Main, Germany; 4 St’Lukes Hospital, Thessaloniki, Greece; 5 Shanghai Gan Ning Medical Technology Inc, Floor 1-2, Building 2, Lane 500, Furonghua Road, Pudong New District, Shanghai, 200135 China; 6 Digital Health Cluster, Hasso Plattner Institut, Potsdam University, Potsdam, Germany; 7 Department of Gastroenterology, Ren Ji Hospital, School of Medicine, Shanghai Jiao Tong University, Shanghai, China; 8 NHC Key Laboratory of Digestive Diseases, Shanghai Institute of Digestive Disease, Shanghai, China; 9 Department of Gastroenterology, Punan Campus, Ren Ji Hospital, School of Medicine, Shanghai Jiao Tong University, Shanghai, China; 10 Department of Infectious Diseases, Southwest Hospital, Third Military Medical University (Army Medical University), Chongqing, China; 11 Center of Integrative Medicine, Beijing Ditan Hospital, Capital Medical University, Beijing, China; 12 Department of Infectious Diseases, Institute of Infection and Immunology, Union Hospital, Tongji Medical College, Huazhong University of Science and Technology, Wuhan, China; 13 Department of Infectious Diseases, Hunan Key Laboratory of Viral Hepatitis, Xiangya Hospital, Central South University, Changsha, China; 14 Department of Hepatology and Infectious Diseases, Second Affiliated Hospital , Chongqing Medical University, Chongqing, China; 15 Department of Infectious Disease, Taihe Hospital, Hubei University of Medicine, Shiyan, China; 16 Department of Hepatology, The First Hospital of Jilin University, Changchun, China; 17 Department of Liver Intensive Care Unit, Shanghai Public Health Clinical Centre, Fudan University, Shanghai, China; 18 Tianjin Institute of Hepatology, Nankai University Second People’s Hospital, Tianjin, China; 19 Infectious Disease Center, The First Affiliated Hospital of Xinjiang Medical University, Urumqi, China; 20 The State Key Laboratory for Diagnosis and Treatment of Infectious Diseases, The First Affiliated Hospital of School of Medicine, Zhejiang University, Hangzhou, China; 21 Department of Infectious Diseases, the Third Affiliated Hospital of Sun Yat-sen University, Guangzhou City, 510630 PR China; 22 Department of Infectious Disease, the First Affiliated Hospital of Xi’an JiaoTong University, Shaanxi, China

**Keywords:** Viral hepatitis, ACLF, Metabolomics, Biomarkers, Nitric oxide, Polyamines, Oxidative stress

## Abstract

**Background/Aims:**

Acute-on-chronic liver failure (ACLF) is one of the deadliest complications of chronic liver disease yet treatment options are sparse. In Asia, HBV-reactivation (HBVr) is one of the most common triggers. Recently, HBV-associated ACLF was associated with distinct changes in the metabolome. Here, metabolic impacts of HBVr were analysed in-depth in non-ACLF, pre-ACLF and ACLF.

**Methods:**

Clinical and metabolic data of 1024 Chinese patients (CATCH-LIFE studies) with chronic HBV mono-infection were analyzed. ACLF was diagnosed according to COSSH criteria. Metabolites in plasma were quantified using LC-MS, compared to non-ACLF and subjected to enrichment and pathway analyses using the database SMPDB via MetaboAnalyst v6.

**Results:**

In 1024 patients (611: non-ACLF, 72: pre-ACLF, 341: ACLF), HBVr was present in 20.2% (ACLF) or 33.3% (pre-ACLF) of patients. HBVr increased 28-day mortality in pre-ACLF patients (2.1% in HBVnr vs. 25% in HBVr). Energy metabolism generating reactive oxygen species (ROS) was highly induced in the absence of ROS-detoxifying pathways in pre-ACLF. Urea cycle, thus nitric oxide (NO) build-up, the polyamine metabolism and related pathways were highly increased in both HBVr pre-ACLF and ACLF, shifting pre-ACLF close to mature ACLF. Specific metabolites could be identified as putative markers and key-regulators herein.

**Conclusion:**

HBV reactivation induces inflammation, hepatic injury and mortality especially in pre-ACLF patients. Metabolic disruption in the ROS/NO/polyamine axis favors stress and hinders liver regeneration. Findings help identifying and alleviating HBVr-driven progression towards ACLF via pharmacological or biopharmaceutical intervention.

**Trial registration:**

Analyses were based on the CATCH-LIFE studies. The CATCH-LIFE studies were registered at Clinical.trials.gov (Clinical Trial Number: NCT02457637, first registered 2015-01-31 and NCT03641872, first registered 2018-08-14).

**Supplementary Information:**

The online version contains supplementary material available at 10.1186/s12964-026-03145-y.

## Highlight

Profound differences in metabolic axes around ROS, NO and polyamines are induced by HBV re-activation in both pre-ACLF and ACLF patients. This shifts pre-ACLF close to mature ACLF and is associated with high inflammation, hepatic insult and mortality. Pathway analyses led to insights into both mode of action and relevant markers. These help to identify diagnostic markers and targets for individualized therapeutic intervention.

**Supplementary Information:**

The online version contains supplementary material available at 10.1186/s12964-026-03145-y.

## Introduction

HBV is an enveloped DNA-virus harboring a circular, partially double stranded 3.2 kb genome, which contains four open reading frames, encoding the Core protein, the secretory HBeAg (HBV e protein), the regulatory HBx (X protein), the viral polymerase and HBsAg (HBV surface antigens) [1]. Acute infections mainly cause transient hepatitis and are rarely accompanied by acute liver failure. Chronic infections, however, are associated with development of liver fibrosis, cirrhosis and associated complications. According to the WHO, the global prevalence of chronically HBV-infected people was around 254 million with a global 1.1 million fatalities linked to HBV-related liver disease and its complications in 2022 [2]. With almost 80 million HBsAg-positive carriers, almost a third of chronically HBV-infected people live in China. In these, highly variable clinical replicative courses are observed. Even though the nomenclature varies between different international societies, the different phases are mainly differentiated on the basis of virological markers as well as clinical and histological characteristics [3, 4]. Accordingly, courses leading to progressive liver disease or being associated with a low or minimal level of inflammation are observed. In any case, HBV persists in liver cells due to the cccDNA (circular covalently closed DNA) pool, in which case HBV is replication-competent in comparison to HBV integrates, which e.g. can express HBsAg. Thus, reactivation of the infection may occur under certain circumstances, even if the immune system has gained functional control over the infection (HBsAg-negative phases).

An acute-on-chronic liver failure (ACLF) is one of the most severe complications of liver disease in which failure of the liver and various other organs can occur. There are several different ACLF definitions and criteria worldwide [5, 6]. The ACLF definition of the Chinese Group on the Study of Severe Hepatitis B (COSSH) is based on the failure of 1 of the 6 major organ systems in a patient with acute decompensation of HBV-related chronic liver disease, with or without cirrhosis [7]. Depending on the number of organ failures and the definition, the 28-day mortality of ACLF varies between 13 and 93% [8]. Therapeutic options for ACLF are strongly limited and rely mostly on treatment of the ACLF inducing and maintaining trigger, on organ-support of the failing organs and liver transplantation if feasible. Pre-ACLF is an episode of acute decompensation, in which an ACLF develops [9]. Although systemic inflammation has been identified as the major underlying driver [10], ACLF courses can be very heterogeneous and therefore specific therapeutic approaches that interrupt the vicious circle are lacking. Whether specific trigger factors cause characteristic disease courses, which would possibly enable therapeutic interventions, is largely unclear. In Europe and North America, the main trigger factors of an ACLF are bacterial infections and alcoholic hepatitis [11]. In contrast, in China and other Asian countries HBVr (HBV reactivation) is the most frequent trigger for development of an ACLF [12]. Nevertheless, little is known about determinants and underlying processes, where initial studies aimed to identify these via profiles of distinct metabolites. In a European study, the systemic inflammation in case of an ACLF was associated with blood metabolite accumulation and profound alterations in major metabolic pathways – in particular inhibition of mitochondrial energy production [13]. In a study from Zhang et al. an HBV-associated ACLF was also associated with distinct changes in the metabolome, including membrane lipid metabolism, steroid hormones, oxidative stress pathways and also energy metabolism [14]. However, little focus was set on relative changes between ACLF and its preceding situation, pre-ACLF, with respect to HBVr as of now.

In this study, we therefore investigated metabolomic characteristics of an ACLF and in how far HBVr acts on the metabolic landscape. Therefore, metabolic data of 1024 Chinese patients from the CATCH-LIFE studies [14] with chronic HBV mono-infection, who were hospitalized due to an acute hepatic exacerbation, were analyzed in regard to HBV-specific metabolic footprints. In order to identify HBV-specific changes in the context of an ACLF, clinical data and the metabolomes of patients diagnosed with HBVr who had no ACLF, pre-ACLF, or ACLF were analyzed and compared to corresponding patients without HBVr (HBVnr).

## Patients and Methods

### Criteria, clinics and ethics

Patients included in this study correspond to the aforementioned CATCH-LIFE study [15], also following the same inclusion and exclusion criteria. Grouping of the cohort was largely applied as described [14] with minor adjustments due to updated diagnostic criteria. Diagnosis of ACLF in cirrhosis adhered to COSSH ACLF criteria [7, 15], thus by the presence of one or more organ failures (liver, coagulation, kidney, cerebral, respiratory or circulation failure) [10]. In non-cirrhosis, ACLF was diagnosed via co-occurrence of liver failure (TB > 10 mg/dl) and coagulation failure (INR > 2.0). An HBV reactivation (HBVr) was defined as either: (i) chronic, HBsAg-positive HBV-infection displaying a more than 2-log increase in HBV DNA levels from baseline, (ii) detection of HBV DNA above a level of 100 IU/mL in patients with undetectable HBV DNA baseline or (iii) a reactivation of a past HBV-infection (HBsAg-negative and antibody against hepatitis B core antigen (anti-HBc) positive) after onset of immunosuppression [12]. Based on these characteristics, the cohort was divided into six groups comprising non-ACLF, pre-ACLF and ACLF patients with (HBVr) and without HBV reactivation (HBVnr). Based on the available data set, pre-ACLF was defined as the development of an ACLF within 28 days for this study, deviating from the EASL definition [9].

Analyses were based on the CATCH-LIFE studies. The CATCH-LIFE studies were registered at Clinical.trials.gov (Clinical Trial Number: NCT02457637, first registered 2015-01-31 and NCT03641872, first registered 2018-08-14). The CATCH-LIFE studies were approved by the ethics committee of the leading center of the CATCH-LIFE studies, Shanghai Jiaotong University School of Medicine, Renji Hospital (No. (2014) 148 k and (2016) 142 k). All patients and volunteers were adequately informed, and written consent was obtained from the study subjects or the legal surrogates of the patients before enrolment. The research was conducted in accordance with the Declaration of Helsinki.

### Data collection, subset generation and enrichment/pathway analyses

Metabolites of patients were collected and analyzed via LC-MS as described previously [14] and in the supplementary methods. Identified, quantified metabolites in pre-ACLF and ACLF patients were compared to non-ACLF patients for HBVr or HBVnr separately to yield log_2_-transformed fold-change in metabolite abundance (LFC) and statistics (Supplementary Table 1). Based on significantly changed metabolites (cutoff: *p* < 0.05), metabolic impact scores were calculated ($\:\mathrm{Metabolic}\:\mathrm{impact}=\stackrel{\sim}{\left|\mathrm{LFC}\right|}\bullet\:\stackrel{\sim}{\left[{-\mathrm{log}}_{10}\left(p\right)\right]}$) and correlated with the number of identified metabolites for each tested group. For final analyses, only a subset of identified metabolites was used (filtered metabolites). Criterion of inclusion was the coverage of metabolites within the database HMDB (Human Metabolome Database [16]) and SMPDB (Small Molecule Pathway Database 2.0 [17]) used for analyses, resulting in *n*=169 (pre-ACLF HBVnr), *n*=185 (pre-ACLF HBVr), *n*=457 (ACLF HBVnr) and *n*=392 (ACLF HBVr) metabolites (Supplementary Table 2). As an effect, this led to a reduction in the absolute numbers of metabolites identified in sera and the number of metabolites used for analyses. Enrichment analyses of filtered metabolites were performed using MetaboAnalyst v6.0 [18] using lipids or metabolites as input selection and SMPDB 2.0 as pathway database. Full metabolite lists (e.g. all ACLF HBVnr) or group-specific metabolite lists (e.g. metabolites unique to ACLF HBVnr not being shared with ACLF HBVr) were used (Fig. 2, Supplementary Fig. 1, Supplementary Table 3) to yield a total of ten enrichment lists including statistics (Supplementary Fig. 1, Supplementary Tables 4–5). Resulting single SMPDB pathway entries were ordered by involvement in overarching metabolic clusters. Entry lists of all involved metabolites were retrieved from SMPDB, which were correlated with metabolites identified in this study to yield pathway coverage ($\:{\mathrm{Cov}}_{path}=\:\frac{\left({n}_{metabolites}\right)\cap\:\left({n}_{SMPDB\_ID}\right)}{{n}_{SMPDB\_ID}}$, with *n*_*metabolites*_ = filtered metabolites and *n*_*SMPDB_ID*_ = metabolites annotated for a given SMPDB pathway entry). These extracted metabolites were then depicted in box and violin plots visualizing the global directional change as determined by the cumulated LFC of all metabolites of a given cluster for each group (refer to Figs. 3 and 4). Alternatively, these were displayed as cumulated, weighted LFC ($\:{\mathrm{L}\mathrm{F}\mathrm{C}}_{W}=LFC\bullet\:\left[{-\mathrm{log}}_{10}\left(p\right)\right]$; refer to Supplementary Fig. 2), as an alternate approach in identifying prominent hits as proposed elsewhere [19]. Lastly, relevant pathways were depicted in a flow diagram with the pathway activity ($\:\mathrm{Pathway}\:\mathrm{activity}={\mathrm{LFC}}_{W}\bullet\:{\mathrm{C}\mathrm{o}\mathrm{v}}_{path}$), which included additional SMPDB pathways as linking nodes (refer to Fig. 5, Supplementary Table 6).

### Statistics and visualization

Clinical parameters were compared amongst different groups by applying a Mann-Whitney U or Chi-square test depending on variable levels. Continuous variables were represented as median (IQR) or mean ± SD according to their distribution, while categorical variables were expressed as counts and percentages. Enrichment analyses were tested for statistical significance within the algorithm of pathway enrichment using MetaboAnalyst v6. Based on a Shapiro-Wilk test, data of global directional change in metabolic clusters was not consistently normally distributed and thus analyzed via a Dunn’s multiple comparisons test (adjusted p-values reported) using GraphPad Prism 10, which was also used to generate graphs. Venn diagrams were generated using Venny 2.1.0 (Oliveros, J.C. (2007–2015)). The pathway flow diagram was partly generated as a vector graphic using Inkscape v1.3.2 (Inkscape Project. (2020). Inkscape. Retrieved from https://inkscape.org).

## Results

### Patient characteristics

**Fig. 1:**
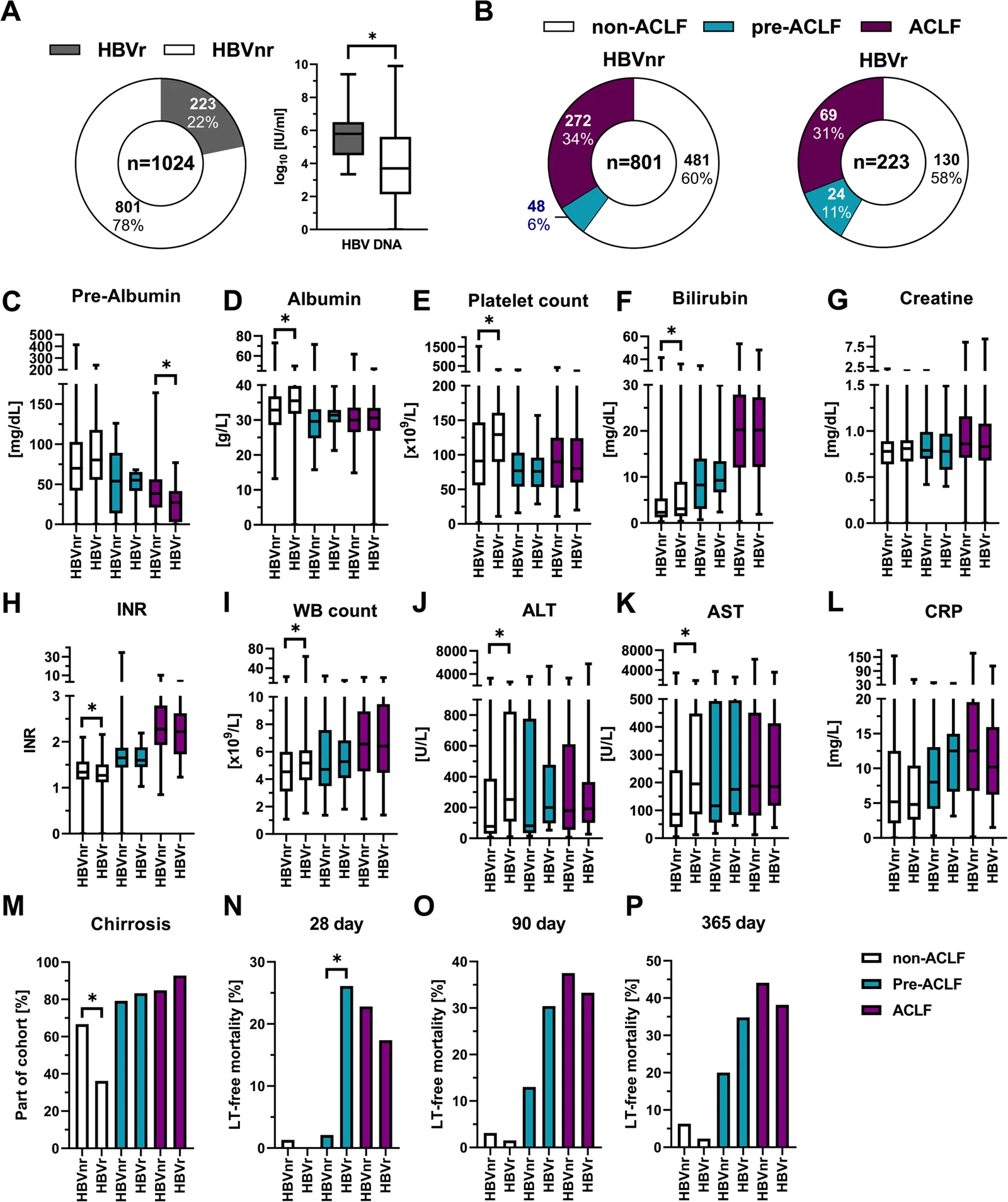
HBV re-activation is accompanied by high basal transaminases and increased mortality in pre-ACLF patients. **A** Parts of cohort with or without HBVr as per viral DNA in IU/mL (international units per mL); n: number of patients. **B** Parts of cohort with non-ACLF, pre-ACLF or ACLF separated by HBVnr and HBVr. **C**-**L** Laboratory markers in sera of patients classified according to panel B; values depicted as mean ± range; INR: international normalized ratio; WBC: white blood cells; ALT: alanine aminotransferase; AST: aspartate aminotransferase; CRP: C-reactive protein. **M** Parts of cohort displaying liver cirrhosis. **N**-**P** LT-free mortality in over 28, 90 or 365 days; LT: liver transplantation. *p<0.05

Overall, 1024 patients from the CATCH-LIFE studies were included in the analyses. Of these, 223 patients were diagnosed with HBVr (HBV reactivation), whereas 801 patients did not fulfil criteria for HBV reactivation and were categorized HBVnr (HBV non-reactivated) (Fig. 1A). ACLF on admission was present in 341 patients (HBVr: *n* = 69; HBVnr: *n* = 272) and significant changes in pre-existing cirrhosis, HBV-DNA, total bilirubin, AST, ALT, albumin, platelet count and haemoglobin were seen upon HBVr. This cohort further was split based on ACLF development within 28 days (pre-ACLF). While pre-ACLF was present in 72 patients (HBVr: *n* = 24; HBVnr: *n* = 48), 611 patients did not fulfil criteria for ACLF or pre-ACLF and were classified as non-ACLF (HBVr: *n* = 130; HBVnr: *n* = 481) (Fig. 1B).

### HBV reactivation leads to increased liver cell damage and mortality in pre-ACLF

 As described above, a total of three main groups were compared in this study: non-ACLF, pre-ACLF and ACLF. These were further divided into six groups based on the presence or absence of HBVr to analyze the interplay between HBVr and ACLF. Initially, we investigated clinical and laboratory parameters (Fig. 1). 

As depicted above, HBVr was characterized by a significant increase in copy number of HBV DNA in patient sera (10^3.39^ vs. 10^5.88^ IU/mL, *p* < 0.05) (Fig. 1A), which was used to split the cohort (Fig. 1B). HBVr was associated with lower prevalence of cirrhosis in the non-ACLF group as compared to HBVnr patients (cirrhosis: 66.7% vs. 36.2%, *p* < 0.05) (Fig. 1M). This goes along with significant higher platelet count and significant higher albumin and total bilirubin in the non-ACLF patients with HBVr (platelet count: 91 vs. 129.5 × 10^9^/L, *p* < 0.05; albumin: 32.85 vs. 35.5 g/L, *p* < 0.05) (Fig. 1D-F). However, higher transaminases and bilirubin were found in non-ACLF patients with HBVr (ALT: 77.9 vs. 252.25 U/L, *p* < 0.05; AST: 86 vs. 194.9 U/L, *p* < 0.05; bilirubin: 2.3 vs. 3.1 mg/dL, *p* < 0.05) (Fig. 1F, J, K). In addition, in non-ACLF a slight, but statistically significant difference was observed in coagulation with lower INR levels in HBVr non-ACLF (1.34 vs. 1.27, *p* < 0.05) (Fig. 1H) and higher white blood count (WBC) (4.53 vs. 5.17, *p* < 0.05) (Fig. 1I). These effects characterize an HBVr in non-ACLF. However, little HBVr-driven effects were seen for pre-ACLF or ACLF. Only pre-albumin was reduced upon HBVr in ACLF (pre-albumin: 38.5 vs. 27 mg/dl, *p* < 0.05) (Fig. 1C). Interestingly, highly relevant HBVr-dependent changes occurred in pre-ACLF with regard to outcome, even if no significant differences were found in the standard laboratory tests within this group. Strikingly, 28-day mortality sharply rose (2.1 vs. 25%, *p* < 0.05) (Fig. 1N) in pre-ACLF upon HBVr, while no statistically significant differences were observed in ACLF and non-ACLF. This makes the HBVr pre-ACLF group the most outstanding and interesting group thus most relevant throughout this manuscript.

### pre-ACLF and ACLF are linked to distinct metabolic pathways in dependency of HBV re-activation.

**Fig. 2:**
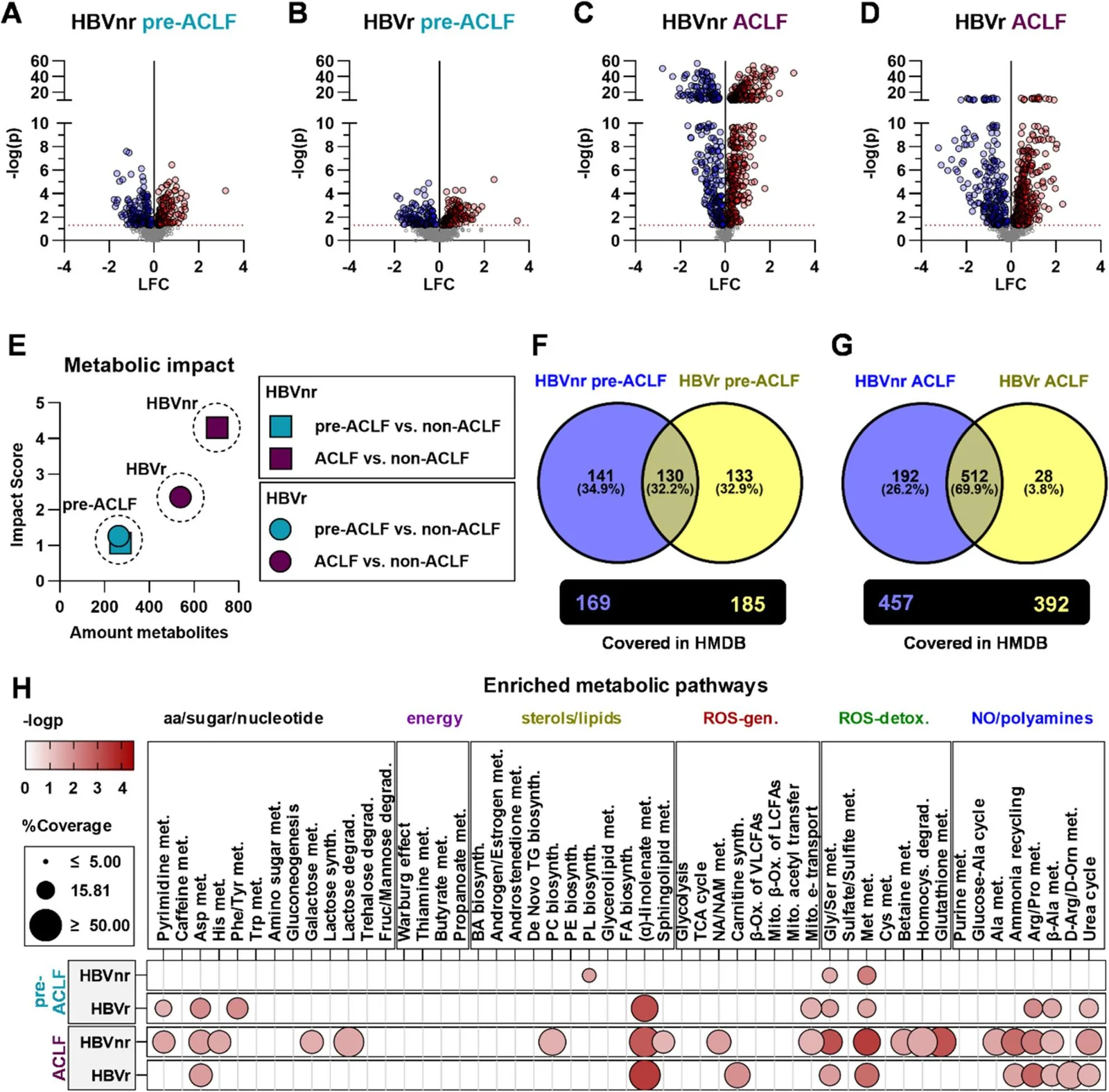
Severity of clinical image correlates with an increase in ROS- and NO-related metabolic pathways. **A**-**D** Identified metabolites as compared to non-ACLF patients; x-axis: log2 fold-change (LFC); y-axis: -log(p), threshold: 1.3); blue dots: less abundant; red dots: more abundant; grey dots: -log(p)<1.3. **E** Metabolic impact of different patient groups as compared to non-ACLF patients (y-axis) correlated to number of metabolites (x-axis); impact factor = Median of absolute LFC multiplied by median of -log(p). **F**-**G** Venn diagrams significant metabolites in panels A-D; numbers in black boxes = metabolites covered by HMDB. **H** Enrichment analyses of metabolites depicted in panels F-G via MetaboAnalyst v6; column titles = metabolic pathways; node color: -log(p) of enrichment (threshold: 1.3); node size: pathways coverage in %; aa: amino acids; ROS: reactive oxygen species; gen.: generation; detox.: detoxification; NO: nitric oxide

 In spite of the steeply increased mortality in pre-ACLF HBVr, no equally apparent clinical or laboratory markers were in place. For a deeper analysis, a metabolomic approach thus was applied with differentially abundant metabolites in plasma samples being subjected to pathway analyses as compared to non-ACLF patients (Fig. 2).

Differentially abundant metabolites clearly increased in number and significance from pre-ACLF to ACLF patients (Fig. 2A-D). Within these groups, 263 (pre-ACLF HBVr), 271 (pre-ACLF HBVnr), 540 (ACLF HBVr) or 704 (ACLF HBVnr) metabolites were significantly changed. While pre-ACLF groups behaved similarly, ACLF groups differed in metabolite count with respect to HBVr. The global metabolic impact (median, absolute, weighted LFC of metabolites) also differed greatly between these groups. Both pre-ACLF groups fell well below ACLF groups, yet HBVr reduced the metabolic impact of an ACLF (Fig. 2E). At first glance, this suggests that HBV does not have a significant impact on pre-ACLF and reduces the severity of mature ACLF. To investigate this further, we performed more in-depth analyses that revealed differences in shared or uniquely deregulated metabolites. Only 4% of metabolites identified in ACLF were unique for HBVr, while 70% were shared with HBVnr and 26% were unique to HBVnr ACLF (Fig. 2G). In contrast, metabolites in pre-ACLF differed strongly with respect to HBVr status giving rise to a tripartite segmentation towards HBVnr-only, HBVr-only or being shared between these (Fig. 2F). This HBVr-based metabolic similarity in ACLF and divergence in pre-ACLF was also visible in metabolic pathway enrichments. For this, either the entirety of significant metabolites in each group (Fig. 2H) or distinct section of Venn diagrams (Fig. 2F, G) were used. Several clusters of metabolic pathways were enriched, with pathways contributing to energy household, ROS (reactive oxygen species) build-up, ROS detoxification or nitric oxide (NO) and polyamine metabolism being dominant. The highest grade of enrichment was found for ACLF HBVnr patients shortly followed by ACLF HBVr. Strikingly, a strong difference between pre-ACLF groups was observed, which stood out from the rest of groups. In HBVr pre-ACLF, the clusters of ROS build-up, ROS detoxification and the NO/polyamine-axis were extremely enriched.

While HBVr dampens the metabolic impact of a mature ACLF, it seemingly shifts the pre-ACLF state closer towards an ACLF as compared to HBVnr, thus worsening the situation. This is most pronounced for metabolic clusters revolving around ROS and NO.

### ROS build-up, ROS detoxification and NO/polyamine metabolism are strongly dependent on ACLF and HBV status

**Fig. 3:**
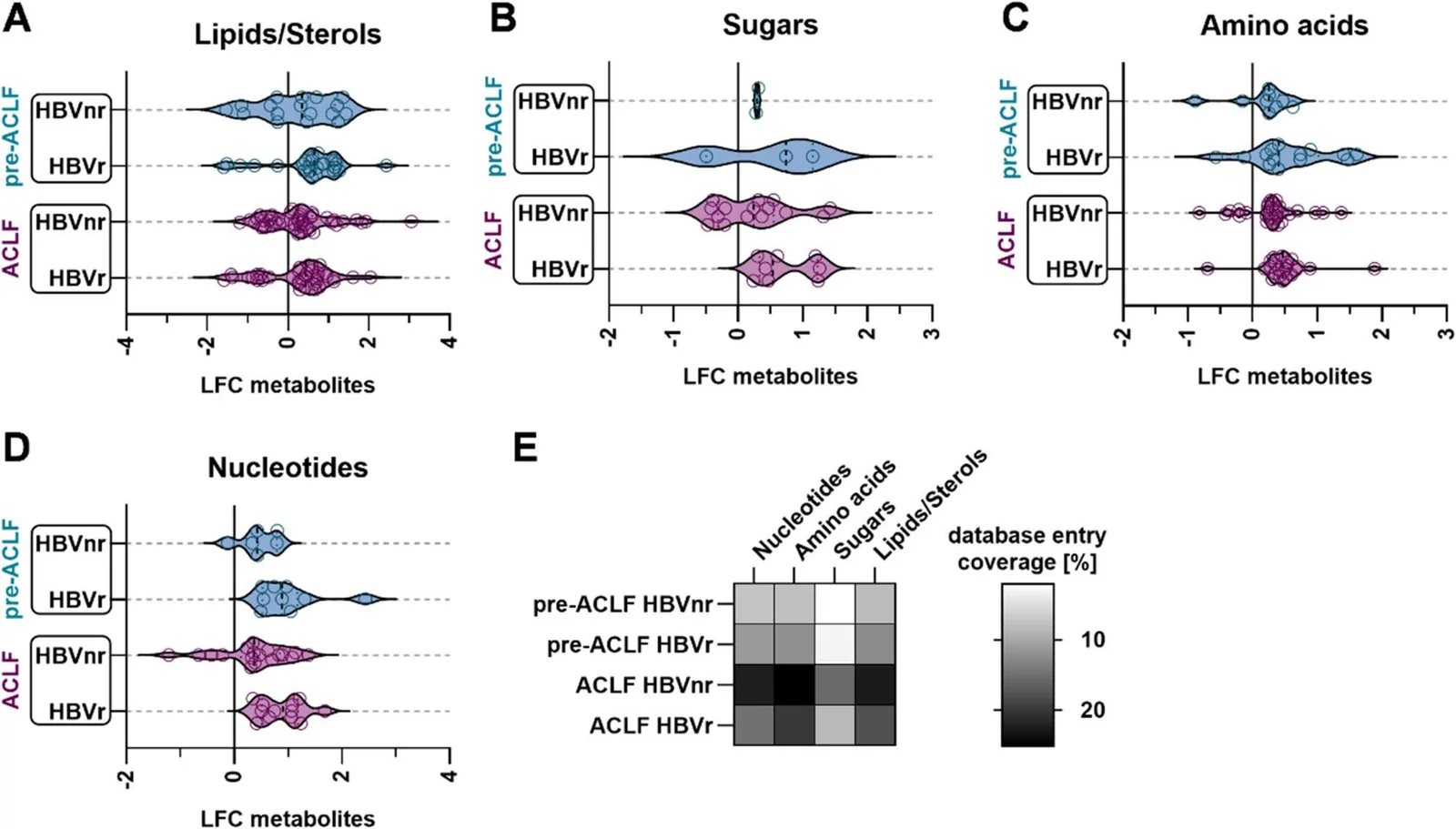
No significant global changes in metabolites of lipids/sterols, sugars, amino acids or nucleotides between different ACLF stages or HBV reactivation statuses. **A**-**D** Violin plots of cumulated metabolites in enriched pathways as compared to non-ACLF patients contributing to lipid/sterol, sugar, nucleotide (pyrimidine and caffeine) and amino acid metabolism for different conditions as of Figure 2H and supplementary Figure 1; x-axis: LFC of metabolites; ***p<0.001, Dunn’s multiple comparisons test. **E** Coverage of combined pathways in panels A-D in %

**Fig. 4:**
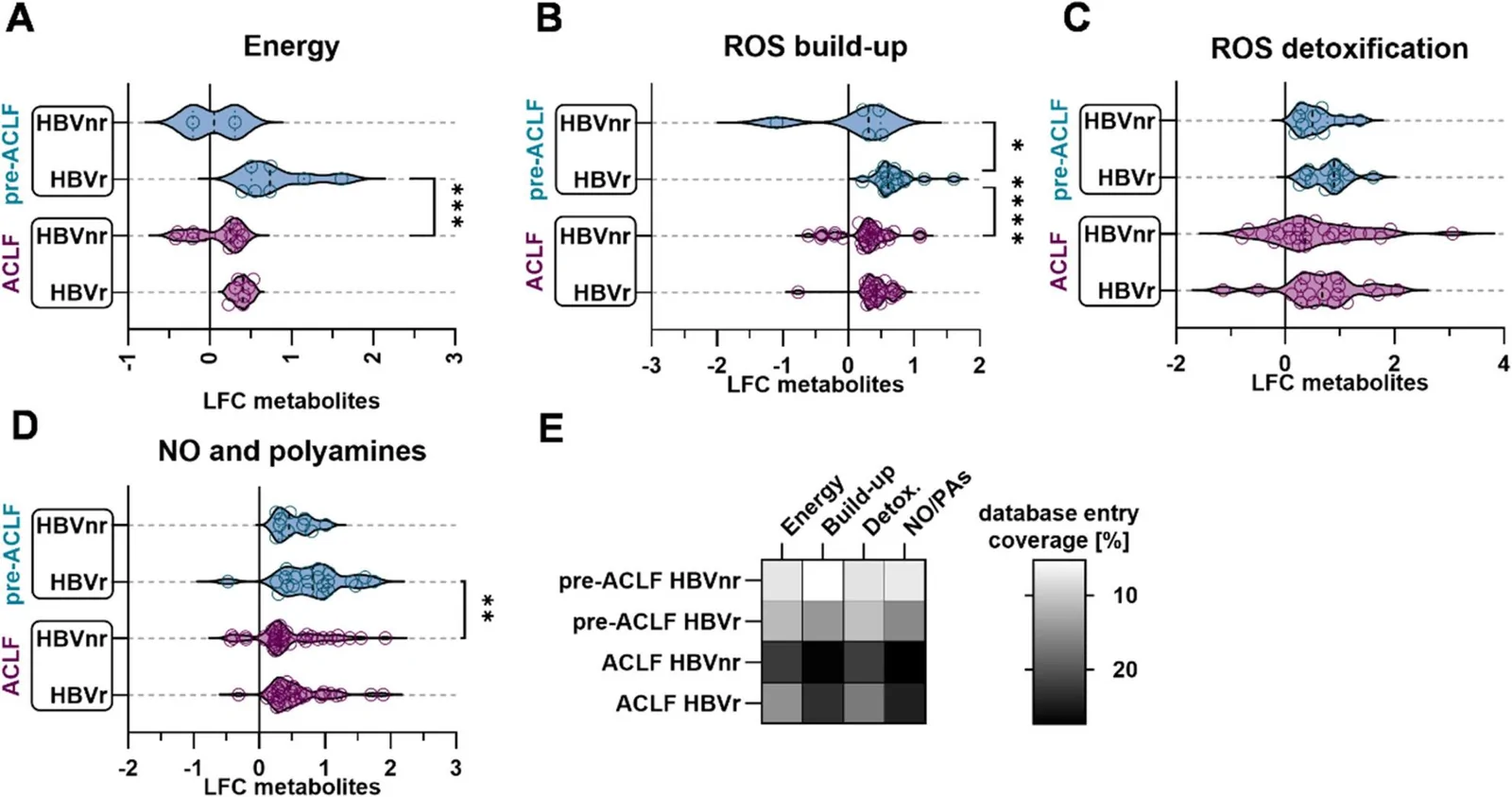
Energy, NO/polyamine metabolism and especially ROS build-up, but not ROS detoxification, is increased in pre-ACLF patients with HBV re-activation. **A**-**D** Violin plots of cumulated metabolites as compared to non-ACLF patients in enriched pathways contributing to energy metabolism, ROS, NO and polyamines for different conditions as of Figure 2H and supplementary Figure 1; x-axis: LFC of metabolites; ROS: reactive oxygen species; NO: nitric oxide. *p < 0.05, **p<0.01, ***p<0.001, ****p<0.0001, Dunn’s multiple comparisons test. **E** Coverage of combined pathways in panels A-D in %

Changes in metabolism around ROS, NO and polyamine metabolism were identified as hallmark of ACLF groups and HBVr pre-ACLF yet not of HBVnr pre-ACLF. As enrichment analyses are non-directional, metabolic clusters were analyzed for whether they are globally up- or downregulated alongside assessing pathway coverage (Figs. 3 and 4). 

a

Generally, lipid/sterol, sugar, nucleotide and amino acid metabolisms were covered best by HBVnr ACLF and least by HBVnr pre-ACLF, with sugar metabolism being the least impactful (Fig. 3E). All metabolic clusters were globally elevated as compared to non-ACLF patients with some metabolites being markedly reduced (Fig. 3A-D). These, in parts, were better resolved by using the weighted LFC (Supplementary Fig. 2A-D). Far scattering metabolites including highly elevated bile acids, steroids, pyrimidines, amino acids or sugars have been identified as promising candidates. On the other side of the spectrum, highly downregulated metabolites such as sphingosine-derivatives, steroids or amino acids have been identified. These were compiled in a list of putative biomarkers and key-regulators (Table 1), which are based on the full list of included metabolites (Supplementary Table 7).

**Table 1:** Far scattering and central metabolites of metabolic clusters lipids, sterols, sugars, amino acids and nucleotides. Metabolites extracted from Fig.3 and Supplementary Fig. 2A-D. LFC as compared to respective non-ACLF group; Weighted LFC = LFC multiplied by -log(p); metabolites listed alphabetically; grey scale as of values for different ACLF stages

Metabolite	LFC				Weighted LFC			
	pre-ACLF		ACLF		pre-ACLF		ACLF	
HBVnr	HBVr	HBVnr	HBVr	HBVnr	HBVr	HBVnr	HBVr	
1.2-dipalmitoyl-GPC (16:0/16:0)	0.35	0.37	0.63	0.59	1.56	1.00	27.03	6.65
1.7-dimethylurate	-	-	-0.66	-	-	-	-1.86	-
1-palmitoyl-GPC (16:0)	-0.44	-	-0.76	-0.76	-1.71	-	-26.05	-7.33
1-palmitoyl-GPE (16:0)	-0.27	-	-0.47	-0.46	-0.63	-	-8.66	-1.86
3-ureidopropionate	0.80	0.55	1.38	1.08	5.13	1.25	59.60	9.22
5.6-dihydrothymine	0.43	0.51	0.71	1.06	1.27	0.98	18.24	13.97
5.6-dihydrouracil	0.43	1.05	0.89	1.24	1.49	4.49	27.77	17.24
5-acetylamino-6-amino-3-methyluracil	-	-	-1.21	-	-	-	-8.94	-
7-HOCA	0.55	0.72	0.96	0.96	2.61	3.10	35.30	11.27
AMP	-	1.61	-	-	-	3.76	-	-
androsterone glucuronide	-1.53	-1.51	-1.19	-1.38	-7.73	-3.97	-15.68	-6.36
arachidonate (20:4n6)	-	0.59	0.39	0.47	-	1.08	3.12	1.41
caffeine	-	-	1.08	1.68	-	-	3.91	4.52
carnosine	-	1.40	0.97	1.88	-	2.50	6.05	9.87
cholesterol	-	-	0.10	-	-	-	0.23	-
citrulline	0.25	0.29	0.41	0.48	0.61	0.51	6.17	2.24
CMP	-	2.43	-	0.75	-	12.66	-	1.10
dehydroepiandrosterone sulfate (DHEA-S)	-	-0.83	-	-0.87	-	-1.39	-	-3.43
estrone 3-sulfate	1.36	-	3.06	2.04	4.32	-	137.06	16.75
etiocholanolone glucuronide	-1.15	-1.60	-0.95	-1.58	-4.05	-4.46	-9.25	-9.31
formiminoglutamate	-	-	1.37	0.88	-	-	35.83	2.56
fructose	-	-0.49	-0.31	-	-	-0.75	-1.00	-
glycochenodeoxycholate	1.22	1.27	1.82	1.13	4.96	3.77	61.27	6.55
glycolithocholate	-	1.16	1.69	0.90	-	2.41	28.73	2.17
hydroquinone sulfate	-	-1.19	-0.61	-1.40	-	-2.10	-2.47	-7.98
indoleacetate	-	-0.57	0.39	-	-	-1.23	2.73	-
inosine 5’-monophosphate (IMP)	-	1.47	-	-	-	3.15	-	-
lactose	-	-	1.32	1.24	-	-	8.43	2.17
mannitol/sorbitol	-	-	0.73	1.19	-	-	6.42	6.94
nisinate (24:6n3)	-1.10	-	-0.61	-0.77	-3.03	-	-1.95	-1.05
orotate	0.33	0.49	0.63	0.65	1.17	1.24	15.35	3.99
S-adenosylhomocysteine (SAH)	0.47	-	1.08	0.70	1.12	-	28.33	2.32
sphinganine	-	1.13	-	-	-	2.38	-	-
sphinganine-1-phosphate	-	-	-0.82	-0.98	-	-	-7.59	-3.37
sphingosine	-	1.17	-	-	-	3.51	-	-
sphingosine 1-phosphate	-0.26	-	-0.69	-0.67	-0.40	-	-11.25	-3.25
stearoyl sphingomyelin (d18:1/18:0)	-0.26	-0.26	-0.58	-0.63	-0.67	-0.53	-23.67	-7.13
sucrose	-	-	1.42	1.26	-	-	14.78	2.63
taurochenodeoxycholate	1.44	1.19	1.93	1.61	5.46	1.91	54.49	10.21
taurocholate	1.24	-	1.37	1.13	2.64	-	14.30	3.06
thymine	0.80	1.31	1.14	1.07	1.99	3.73	16.75	3.99
tyrosine	0.25	0.40	0.45	0.51	0.55	1.39	11.67	4.97
uridine	-0.12	-	-	-	-0.18	-	-	-
xanthurenate	-0.89	-	-0.82	-0.70	-2.31	-	-7.87	-1.84

While these metabolites certainly are interesting, they did not explain differences seen between ACLF stages or HBVr, as clusters did not differ relatively. This was different in ROS- and NO/polyamine-metabolic axes (Fig. 4).

The coverage of these clusters was again greatest in HBVnr ACLF and least in HBVnr pre-ACLF (Fig. 4E), which may be due to a larger sample size. However, both HBVr groups were found to be very close to HBVnr ACLF rather than HBVnr pre-ACLF, which only had little coverage overall and especially for ROS build-up and NO/polyamine metabolism. This stood in contrast to the pre-ACLF HBVr group, indicating HBVr triggering a shift towards an ACLF. Most importantly, the HBVr pre-ACLF also stood out significantly with respect to directional change. While the majority of metabolites were upregulated in general, HBVr significantly upregulated energy metabolism (26.14 vs. 9.857; z = 3.996), ROS build-up (61.29 vs. 30.03; z = 4.483) and NO/polyamine metabolism (71.05 vs. 43.62; z = 3.346) in pre-ACLF (Fig. 4A-D). Noteworthy, this was not accompanied by a relative upregulation of ROS detoxification (Fig. 4C). From these clusters, some key metabolites could be extracted by using the weighted LFC (Supplementary Fig. 2E-H). Far-scattering, highly increased metabolites comprised redox-competent metabolites or precursors of these, energy and redox-related nucleotide-derivatives or metabolites of the urea cycle and polyamine synthesis. Highly reduced metabolites were less abundant yet comprised links to the redox-system or the urea cycle. These and central key-metabolites have been extracted (Table 2) from the full list of included metabolites (Supplementary Table 7).

**Table 2:** Far scattering and central metabolites of metabolic clusters energy metabolism, ROS build-up, ROS detoxification and NO/polyamine metabolism. Metabolites extracted from Fig. 4 and Supplementary Fig. 2E-H. LFC as compared to respective non-ACLF group; Weighted LFC = LFC multiplied by -log(p); metabolites listed alphabetically; grey scale as of values for different ACLF stages

Metabolite	LFC				Weighted LFC			
	pre-ACLF		ACLF		pre-ACLF		ACLF	
HBVnr	HBVr	HBVnr	HBVr	HBVnr	HBVr	HBVnr	HBVr	
1-methylnicotinamide	-	-	0.69	0.49	-	-	10.93	1.03
2-oxoarginine	0.32	-	0.72	0.47	0.70	-	12.80	1.63
3-phosphoglycerate	-	1.15	-	-	-	2.48	-	-
3-ureidopropionate	0.80	0.55	1.38	1.08	5.13	1.25	59.60	9.22
5.6-dihydrouracil	0.43	1.05	0.89	1.24	1.49	4.49	27.77	17.24
5-methylthioadenosine (MTA)	1.01	-	1.54	0.98	3.50	-	33.45	3.10
acetylcarnitine (C2)	-	-	0.41	0.55	-	-	3.12	2.43
adenine	0.69	-	1.20	0.62	1.90	-	19.59	1.08
alpha-ketoglutarate	-	0.74	-	-	-	1.03	-	-
AMP	-	1.61	-	-	-	3.76	-	-
arachidonate (20:4n6)	-	0.59	0.39	0.47	-	1.08	3.12	1.41
arginine	0.25	-	0.36	0.52	0.67	-	4.23	3.01
betaine	0.30	0.38	0.87	0.64	0.63	0.65	33.41	4.50
carnosine	-	1.40	0.97	1.88	-	2.50	6.05	9.87
citrate	0.31	-	-0.08	-	0.46	-	-0.12	-
citrulline	0.25	0.29	0.41	0.48	0.61	0.51	6.17	2.24
cystathionine	0.69	0.95	1.36	1.12	2.26	2.78	53.75	8.45
cysteine	0.18	-	0.54	0.44	0.24	-	11.64	2.12
cysteinylglycine	-	-	1.09	0.89	-	-	10.69	2.07
dimethylglycine	-	-	-0.68	-0.49	-	-	-12.18	-1.88
estrone 3-sulfate	1.36	-	3.06	2.04	4.32	-	137.06	16.75
FAD	-	0.74	0.36	0.34	-	1.70	1.72	0.46
fumarate	-	0.40	0.26	0.40	-	0.56	0.97	1.12
glucose	-	-	-0.40	-	-	-	-1.51	-
guanidinoacetate	0.31	0.42	0.80	0.95	0.66	0.59	18.45	8.65
heme	-	0.89	-	-	-	1.28	-	-
inosine 5’-monophosphate (IMP)	-	1.47	-	-	-	3.15	-	-
linolenate (18:3n3 or 3n6)	-	0.85	0.54	0.67	-	2.57	5.56	2.41
malate	-	0.55	0.35	0.47	-	1.39	3.02	2.51
methionine	0.67	0.89	1.92	1.70	2.73	2.11	96.27	19.72
methionine sulfoxide	0.53	0.92	1.74	1.54	1.94	2.47	90.71	18.54
nicotinamide	-	0.70	-	0.45	-	1.24	-	1.11
nicotinamide riboside	0.51	-	1.09	0.65	0.81	-	16.84	1.31
nisinate (24:6n3)	-1.10	-	-0.61	-0.77	-3.03	-	-1.95	-1.05
ornithine	-	0.42	0.24	0.27	-	1.34	1.31	0.59
phenol sulfate	-	-	-0.80	-1.14	-	-	-4.70	-5.45
pyruvate	-	-	0.24	-	-	-	0.32	-
S-adenosylhomocysteine (SAH)	0.47	-	1.08	0.70	1.12	-	28.33	2.32
spermidine	-	1.07	0.70	0.94	-	2.51	6.66	3.72
spermine	-	1.74	-	-	-	4.58	-	-
urate	-	-0.48	-0.44	-0.32	-	-2.37	-7.92	-1.27
urea	-	-	-	0.31	-	-	-	0.48
xanthine	-	0.71	0.37	0.28	-	1.40	2.60	0.68
xanthosine	-	0.96	1.20	1.17	-	1.85	19.06	5.50

In summary, a deep analysis of metabolic clusters revealed putative markers for different stages of ACLF and the role of HBVr therein. However, analyzing the metabolic clusters around ROS- and NO-related metabolism revealed that HBVr is metabolically shifted from the pre-ACLF group towards an ACLF. Consequently, ROS build-up and NO/polyamine metabolism, but not ROS detoxification, is the most augmented in this group of patients.

### HBV reactivation drives mitochondrial ROS-generation in the absence of ROS-detoxification in pre-ACLF patients

**Fig. 5:**
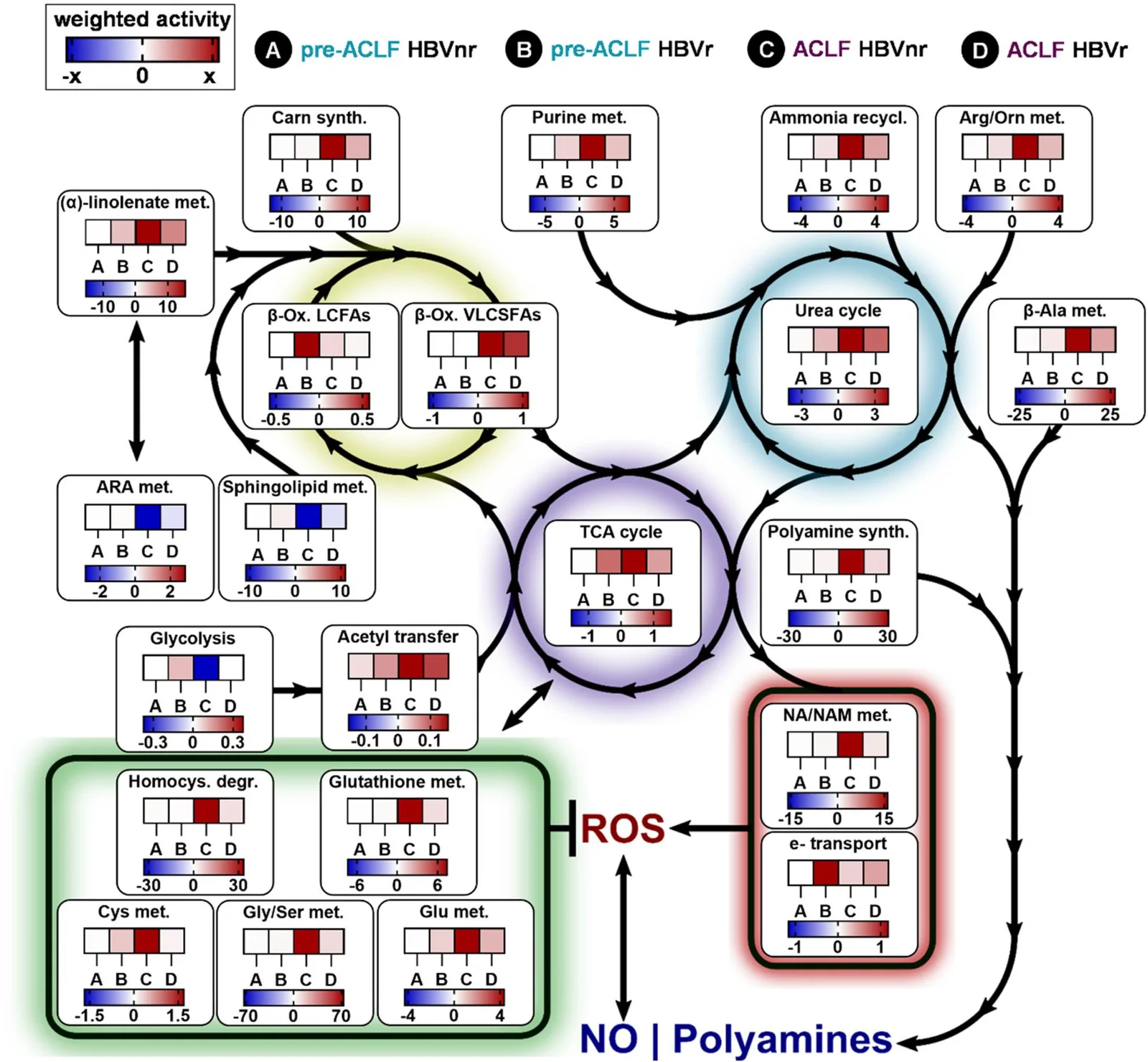
Impacted pathways revolve around mitochondrial metabolism impacting ROS-NO-polyamine axis. Integrative pathway network of selected enriched pathways and additional ones (ARA, Polyamine synth.) assembled by literature search; coloring: weighted pathway activity; met.: metabolism; synth.: synthesis; recycl.: recycling; degr.: degradation; Carn: carnitine; Arg: arginine; Orn: ornithine; β-Ala: beta alanine; Cys: cysteine; Gly: glycine; Ser: serine; Glu: glutamate; TCA cycle: citrate cycle; (α)-linolenate: linoleic acid and alpha-linolenic acid; ARA: arachidonic acid; Homocys.: homocysteine; acetyl transfer: mitochondrial acetyl transfer; β-Ox.: beta oxidation; LCFAs: long chain fatty acids; VLCSFAs: very long chain saturated fatty acids; e- transport: mitochondrial electron transport; NA/NAM: nicotinate and nicotinamide

To combine aforementioned insights, an integrative pathway map was generated. This includes all respective pathways of clusters in Fig. 2H and Supplementary Fig. 1 as well as additional pathways based on literature and a directed search in the HMDB database. Here, pathway coverage, metabolite LFC and significance were combined to yield a single projection value of a weighted pathway activity (Fig. 5).

ACLF presented as the harshest condition activating pathways with HBVr inducing a dampening effect. This held true for the module around β-oxidation, the TCA cycle or nicotinamide metabolism. Fittingly, this went along with a reduction in glycolysis. This increase in mitochondrial metabolism went in line with the greatest increase in ROS-detoxifying systems or pathways related to these. While polyamine synthesis and generation of NO were most active in ACLF HBVnr, systems linked to inflammatory modulation (arachidonic acid metabolism and sphingolipid metabolism) were strongly reduced. Notably, on the route from ROS-generation over ROS-detoxification to regenerative pathways, HBVnr pre-ACLF was very distinct from both mature ACLF groups and most importantly to HBVr pre-ACLF. In this group, important dysregulations were in place especially in glycolysis, β-oxidation of long chain fatty acids and, most importantly mitochondrial electron transport as the primary source of superoxide, is highest in the pre-ACLF HBVr group. This went along with no major increase in prime ROS-detoxifying systems and only a minor increase in regenerative metabolism. Lastly, pre-ACLF HBVr was the sole group displaying a visible induction in the metabolism of inflammatory modulators.

Taken together, there is a fundamental difference from both mature ACLF groups and most importantly from the HBVr pre-ACLF group to pre-ACLF HBVnr, which is a strong induction of β-oxidation- and TCA cycle-driven increase in mitochondrial electron transport, thus in ROS-generation. This is not counteracted by ROS-detoxifying systems or majorly compensated through an induction of regenerative processes.

## Discussion

Our study presents deep and novel insights into the metabolic regulation of not only a pre-ACLF versus an ACLF, but especially regarding the extent to which HBVr accounts for relative changes herein. It gives rise to profound changes in clinical outcomes, especially in 28-day mortality being more than 10-fold increased in HBVr pre-ACLF as compared to HBVnr pre-ACLF. Surprisingly, no apparent, equally pronounced change in routinely used clinical markers is in place in pre-ACLF, whereas significant differences are observed in non-ACLF patients in regard to transaminases. This may, in parts, reflect an overall increased hepatic insult in HBVr or maybe its consequence. Also, a reduction in metabolic capacity, as for a decrease in pre-albumin levels in ACLF HBVr, is observed, which is a robust marker of HBV-ACLF [20]. However, focusing only on these parameters does come with some ambiguities: (i) HBVr strongly increases mortality in pre-ACLF only, (ii) HBVr goes along with an increased hepatic injury only in non-ACLF and (iii) HBVr impairs metabolic capacity of the liver only in ACLF. Thus, an expansion of parameters is required in an aim to pinpoint both key metabolites and mechanisms, which we used metabolomics for. In this precise setting, performing metabolomics sees one key advantage over other omics approaches such as proteomics or transcriptomics, which is sample availability. Plasma samples were thus used as projective markers for tissue status, such as liver metabolism, as other approaches require more invasive and laborious techniques to generate biopsies and analytes.

Considering the global metabolic impact between the groups (HBVr vs. HBVnr), two surprising key findings were made. Firstly, HBVr dampened the metabolic impact of an ACLF. We explain this phenomenon with the overall decrease in hepatic fitness: a higher degree in hepatic insult results in reduced metabolic capacity due to the lack of functional tissue. Secondly, there is no relative difference in metabolic impact between pre-ACLF groups, although mortality is dramatically different. However, the metabolic impact score, as used herein, represents a composite metric derived from the median absolute log2 fold-change weighted by statistical significance. While this approach allows a concise, group-level comparison of overall metabolic perturbation, it is inherently insensitive to directional heterogeneity and does not discriminate between pathway-specific upregulation and downregulation [19]. Therefore, directional cluster analyses were performed.

While mature ACLFs share large parts of deregulated metabolites, pre-ACLF groups are well distinguishable from each other in terms of identified metabolites thus suggesting the mortality being driven by these. These differential metabolites are enriched in dominant clusters: (i) ROS-generation, (ii) ROS-detoxification and (iii) NO-generation and polyamine metabolism. Here, HBVr clearly led to pre-ACLF resembling footprints of an ACLF. Interestingly, this did not manifest in the overall metabolic footprint, but in these precise clusters. While other clusters may see relevance for ACLF in general, they do not appear to contribute to HBVr as for the absence of relative differences to HBVnr. Interestingly, this also included the majority of sphingolipids, which in parts differentiated between non-ACLF, pre-ACLF and ACLF in our dataset as described before [21, 22], but not between HBVr therein. The HBVr-induced mortality therefore seems to be strongly associated to metabolic deregulations in ROS/NO/polyamine clusters. This correlation suggests that, when excessively activated, all three pathways shift from protective signaling to cell- and tissue-damaging stress. For example, elevated ROS levels can directly cause oxidative damage to lipids. Furthermore, high ROS levels can trigger cell death pathways, such as oxeiptosis, which is a ROS-triggered, caspase-independent cell death pathway [23], leading to tissue injury and inflammation. Excess NO can combine with ROS, thereby exacerbating oxidative damage and inflammatory injury. Furthermore, deregulated NO levels may affect the control of vasodilation. Apart from its direct impact on the liver, deregulated polyamine metabolism is implicated in renal failure [24].

Metabolites of energy metabolism and mitochondrial ROS-generating pathways are globally highest in HBVr pre-ACLF – even exceeding mature HBVnr ACLF. There is a clear induction of mitochondrial acetyl transfer and β-oxidation as well as the TCA cycle. Consequently, mitochondrial electron transport in the respiratory chain is highest in HBVr pre-ACLF. Inevitably, ROS are generated therethrough and no matching increase in ROS-detoxification systems is in place. With respect to this, protective cystathionine levels [25] are increased in all groups. However, the glutathione-dependent detoxification system is highly induced in HBVnr ACLF. This system is dampened by HBVr in ACLF, which is even more pronounced in HBVr pre-ACLF, where it is lowest. In line with this, this system is reported to be downregulated on a metabolic level by HBV in vitro [26], although the Nrf2-ARE (nuclear factor erythroid 2-related factor 2 / antioxidant response element) system is induced by HBV [27] – an ambiguity which may be due to functionality of the host cells or tissue. A second prominent route of superoxide-production is presented by the purine metabolism-resident XO (xanthine oxidase) [28], with xanthine being increased in all but the HBVnr pre-ACLF group. However, urate, the product of xanthine conversion, is highly reduced in these groups. In effect, ROS generated at the respiratory chain and not via XO is relevant. Mitochondrial ROS-production likely is increased through the onset of the HBV life cycle in HBVr. Especially HBx is known to induce mitochondrial dysfunction [29] in a genotype-specific fashion [30], which in turn promotes viral gene expression [31]. Although this triggers an antioxidant response via the Nrf2/ARE system [32], oxidative stress prevails and contributes to disease progression [27]. This then loops back to the hepatic insult being increased by inflammation and ROS and to loss of metabolic tissue and an energy deficit, as reflected by high AMP-levels only in HBVr pre-ACLF. As a stimulus of catabolism [33], glycolysis and mitochondrial systems are increased further generating ROS. This cycle is worsened by the HBx-driven mitochondrial dysfunction, making oxidative phosphorylation inefficient as reflected by main metabolites of the TCA cycle (α-ketoglutarate, fumarate, malate, FAD) being elevated only in clinically noticeable and high-mortality groups and not in HBVnr pre-ACLF, which has been reported in different settings [34]. Also, an increase in ROS could see relevance in the regulation of sphingomyelins/ceramides, which is correlated with ACLF [21]. Here, S1P (sphingosine-1-phosphate) acts as physiological counterplayer to ceramides [35], which induce ROS. Just as described before [36], we observe a steep reduction in S1P, which could contribute to ceramides overtaking the equilibrium thus favoring ROS-production.

In a functioning system, the hepatic insult would, in parts, be compensated by liver regeneration. One dual role herein is fulfilled by NO. It can act both as radical scavenger and inducer of oxidative stress [37]. Also, strong interactions with mitochondrial and homocysteine-based ROS build-up exist [38]. Notably, homocysteine, proposed as promising marker for HBV-ACLF [39], induces mitochondrial dysfunction [40] yet seems to be reduced as for the upregulation of homocysteine degradation in both HBVnr and HBVr ACLF. With respect to damage compensation, NO is classically seen as contributor to liver regeneration [41]. A prime source of NO is found within the conversion of arginine to citrulline via NOS (nitric oxide synthase) as part of the urea cycle, with the latter being increased in all groups of the cohort. Going further, differences in associated pathways can be seen with respect to purine metabolism, ammonia recycling and β-alanine metabolism. These pathways also provide precursors for polyamine synthesis, a second major set of liver-regenerative metabolites [42]. Here, the precursor ornithine and the polyamine spermidine are highly increased in all groups but HBVnr pre-ACLF, with spermine only being increased in HBVr pre-ACLF and putrescine not being elevated in any. Regenerative markers therefore are in place yet the increase in pathogenesis speaks against an effective regeneration. One interpretation could be an increased polyamine level being due to an accumulation of those not being consumed for regeneration. Alternatively, absence of regeneration could also be due to the harshly upregulated generation of ROS. An example for this is the ROS-dependent insulin-resistance, a key-regulator of liver regeneration [43], e.g. via JNKs (c-Jun N-terminal kinases ) [44]. This similarly is a hallmark of HBV [45, 46] thus adding up to the situation. An increase in NO-generation seemingly would be beneficial as for its essential role at least in case of NO originating e.g. from iNOS (inducible nitric oxide synthase) [47]. However, it can also worsen symptoms in case of decompensated end-stage liver disease [48] thus being considered as marker for high- and low-risk ACLF [49]. Further, increased NO concentrations could influence the complex hemodynamic interplay between portal hypertension on the one hand and systemic hypotension in ACLF on the other hand. On the side of HBV, HBx may again be central as an inducer of iNOS [50], which would pair with the HBV-dependent increase in metabolites of the urea cycle in its natural course of infection [51] and thus also substrates for NOS. Lastly, while polyamines would provide for an increase in liver regeneration, they also boost HBV replication as pointed out recently [52]. Consequently, both regenerative markers may actually worsen the clinical outcome of patients in this specific case.

In this study, we observed a dramatically increased mortality of pre-ACLF patients in HBVr. This observation is based on a single cohort, so it will be interesting to investigate this in other cohorts, for example from other regions of the world. For further mechanistic insights we identified novel factors contributing to this clinical picture. Through metabolomics, we correlated HBVr with augmented oxidative stress and NO/polyamine metabolism at distinct stages. This facilitates selecting putative biomarkers in the future, a list of which was provided in our study. Promising candidates include the strongly differentially dysregulated carnosine, CMP, linolenate, 5,6-dihydrouracil/thimine, methionine, urate, xanthurenate and spermidine. However, all candidates identified in this observational study require prospective validation in independent cohorts before any diagnostic application can be considered. Further, the pathways may be used for interventions. In the future, stabilizing mitochondrial systems, modulating the urea cycle and associated pathways and shifting polyamine equilibrium should be in the focus of HBV-ACLF prevention according to our data. This could be achieved via classical, pharmacological intervention, and possibly in the future via gene-therapy, novel biomedicines such as therapeutic antibodies and also by targeting HBV via a therapeutic vaccine.

## Limitations

Several limitations of this study warrant consideration. First, all analyses were performed within a single Chinese cohort (CATCH-LIFE studies), which limits the generalizability of the findings. Second, the pre-ACLF HBVr group comprised only 24 patients, and while effect sizes were reported alongside p-values to account for this, conclusions drawn from this subgroup should be interpreted with appropriate caution. Third, the substantially different sample sizes between the ACLF HBVr (*n* = 69) and ACLF HBVnr (*n* = 272) groups represent a potential confounding factor, as lower statistical power in smaller groups may result in fewer metabolites reaching significance independently of true biological differences. Of note, the observed pattern of qualitative pathway overlap with quantitative attenuation of effect sizes across the ACLF groups argues against this being the sole explanation; nonetheless, this imbalance should be acknowledged when interpreting group comparisons. Fourth, as this is an observational, cross-sectional metabolomic study, causal inferences cannot be drawn from the associations reported.

## Supplementary Information

Supplementary Material 1.

## Data Availability

Calculations, datasets and generated lists are fully published alongside the manuscript on Mendeley Data, which can be accessed via the following link as a preview during the revision process: (https://data.mendeley.com/datasets/89nrz3bbh9/1). The corresponding DOI is 10.17632/89nrz3bbh9.1. Further information can be made available upon request.

## Abbreviations

ACLF: Acute-on-chronic liver failure
ALT: Alanine aminotransferase
AMP: Adenosine monophosphate
AMPK: AMP-activated protein kinase
AST: Aspartate aminotransferase
CATCH-LIFE: Chinese Acute-on-Chronic Liver Failure study
cccDNA: Circular covalently closed DNA
CMP: Cytidine monophosphate
CRP: C-reactive protein
COSSH: Chinese Group on the Study of Severe Hepatitis B
DHEA-S: Dehydroepiandrosterone sulfate
DNA: Deoxyribonucleic acid
EASL: European Association for the Study of the Liver
FAD: Flavin adenine dinucleotide
HBcAg: Hepatitis B core antigen
HBeAg: Hepatitis B e antigen
HBsAg: Hepatitis B surface antigen
HBV: Hepatitis B virus
HBVnr: HBV non-reactivated
HBVr: HBV reactivation
HBx: Hepatitis B virus X protein
HMDB: Human Metabolome Database
IMP: Inosine 5’-monophosphate
INR: international normalized ratio
IQR: Interquartile range
JNK: C-Jun N-terminal kinase
LC-MS: Liquid chromatography–mass spectrometry
LFC: Log2 fold-change
LT: Liver transplantation
MTA: 5-methylthioadenosine
NO: Nitric oxide
Nrf2: Nuclear factor erythroid 2-related factor 2
ROS: Reactive oxygen species
SAH: S-adenosylhomocysteine
SMPDB: Small Molecule Pathway Database
TB: Total bilirubin
WBC: White blood cell count
WHO: World Health Organization
