# Supplementary material for: HBV re-activation is associated with increased mortality and dysregulation of oxidative and polyamine metabolism in pre-acute-on-chronic liver failure
Source: Cell Commun Signal. 2026 Aug 24;24:460. doi: 10.1186/s12964-026-03145-y (PMC13508325; doi:10.1186/s12964-026-03145-y)
Supplement: Supplementary file 1 [file 12964_2026_3145_MOESM1_ESM.docx]

# Supplementary information

**Supplementary methods:**

**Metabolomic profiling**

The metabolomics profiling was done at Calibra Diagnostic Company (Hangzhou, China), which operated an untargeted Discovery HD4 metabolomics platform from Metabolon (Durham, North Carolina, USA) as described [1].

Briefly, 100 µl of serum per sample underwent protein precipitation with methanol, followed by centrifugation, and the resulting supernatant was divided into four equal aliquots. Each aliquot was subjected to a distinct ultra-high-performance liquid chromatography coupled to tandem mass spectrometry (UHPLC-MS/MS) acquisition method: two aliquots were measured under acidic positive ion mode electrospray ionization (ESI) conditions using a reverse-phase C18 column (Waters ACQUITY BEH C18, 2.1 × 100 mm, 1.7 µm), with one method optimized for more hydrophobic analytes; a third aliquot was analyzed under basic negative ion mode ESI conditions using the same column chemistry; and a fourth aliquot was measured in negative ion mode using a hydrophilic interaction liquid chromatography (HILIC) column (Waters ACQUITY BEH Amide, 2.1 × 150 mm, 1.7 µm). All methods were run on a Waters ACQUITY UPLC system coupled to a Thermo Scientific Q-Exactive high-resolution mass spectrometer equipped with a heated ESI source (HESI-II), alternating between full-scan MS and data-dependent MS/MS acquisition with dynamic exclusion. Metabolite identification relied on matching to an in-house library of authentic standards using retention time, exact mass-to-charge ratio (m/z), and MS/MS fragmentation spectra. Peak areas of primary MS ions were used for quantification, and inter-batch normalization was performed using the median signal of a pooled quality control (QC) sample, which was inserted at regular intervals throughout each analytical run.

**Supplementary figures:**

**Supplementary Figure 1:** **Severity of clinical image correlates with an increase in ROS- and NO-related metabolic pathways.** Enrichment analyses of metabolites and subsection of Venn diagrams depicted in panels F-G via MetaboAnalyst v6; column titles = metabolic pathways; node color: -log(p) of enrichment (threshold: 1.3); node size: pathways coverage in %; aa: amino acids; ROS: reactive oxygen species; gen.: generation; detox.: detoxification; NO: nitric oxide.

**Supplementary Figure 2:** **Identification of putative metabolic markers in metabolic clusters using the weighted LFC of metabolites.** (**A-H**) Violin plots of cumulated metabolites in enriched pathways contributing to lipid/sterol, sugar, nucleotide (pyrimidine and caffeine), amino acid, ROS, NO, polyamine metabolism for different conditions as of Figure 2H and supplementary Figure 1; x-axis: weighted LFC as calculated by multiplying LFC by corresponding negative log(p); ROS: reactive oxygen species; NO: nitric oxide; *p<0.05, Dunn’s multiple comparisons test.

## 1. Supplementary table legends

Supplementary tables can be retrieved from the URL to Mendeley Data provided in the data availability statement.

**Supplementary Table 1: Entire list of identified metabolites for different groups.** Data split into supergroups referring to HBVr (HBV reactivation) or HBVnr (no HBVr) and pre-ACLF and ACLF; comparisons to abundance of metabolites in respective non-ACLF groups; different classifications of metabolites given in form of biochemical name, super-pathway, sub-pathway, chemical identifier (ID), refractive index, monoisotopic mass, Pubchem ID, CAS number, KEGG ID and HMDB ID; statistics of comparisons to non-ACLF group given in form of FDR (false discovery rate), negative decadic logarithm of p-value (p), fold-change (FC), log2 FC (logFC); significant metabolites marked in red.

**Supplementary Table 2*:* List of metabolites used for downstream analyses.** Data represents filtered and condensed list of metabolites given in Supplementary Table 3; only significant metabolites (-log(p)>1.3) and those having an HMDB entry included; weighted LFC corresponds to LFC (logFC) multiplied by -log(p).

**Supplementary Table 3: Shared and unique metabolites for different groups.** Data refers to Venn diagrams for either pre-ACLF or ACLF subgroups depicted in Figure 2. HMDB ID refers to metabolites of intersections in Venn diagrams, which were listed alongside their LFC, statistics and weighted LFC in Supplementary Table 4.

**Supplementary Table 4: Identified pathways by enrichment analyses on metabolites of pre-ACLF subgroups.** Lists of metabolites used as input for enrichment analyses are given in Supplementary Table 3; five different enrichment analyses were performed on pre-ACLF groups according to the Venn diagram in Figure 2. Table returns pathway name (Metabolite set), number of entries included in the database entry (Total), number of intersections with the input data (hits), enrichment statistics (p-value, Holm P, FDR and -log(p), coverage as assessed by comparing hits and total (%coverage); Condensed enrichment tables combine relevant data used for depiction.

**Supplementary Table 5: Identified pathways by enrichment analyses on metabolites of ACLF subgroups.** Lists of metabolites used as input for enrichment analyses are given in Supplementary Table 3; five different enrichment analyses were performed on ACLF groups according to the Venn diagram in Figure 2. Table returns pathway name (Metabolite set), number of entries included in the database entry (Total), number of intersections with the input data (hits), enrichment statistics (p-value, Holm P, FDR and -log(p), coverage as assessed by comparing hits and total (%coverage); Condensed enrichment tables combine relevant data used for depiction.

**Supplementary Table 6: SMPDB pathways and metabolites used to generate a pathway map.** Table returns all included metabolites for respective metabolic pathways depicted in Figure 5. Long name, short name and SMPDB ID give information on which pathway was presented in graphs displayed in Figure 5 in form of dot-plots; HMDB IDs refer to metabolites being part of the respective SMPDB IDs and were used to extract values of metabolite LFC and statistics.

**Supplementary Table 7: Full list of metabolites of clusters lipids, sterols, sugars, amino acids and nucleotides.** Extracts displayed in Figure 3 and Supplementary Figure 2A-D. LFC as compared to respective non-ACLF group; Weighted LFC = LFC multiplied by -log(p).

**Supplementary Table 8: Far scattering and central metabolites of metabolic clusters energy metabolism, ROS build-up, ROS detoxification and NO/polyamine metabolism.** Extracts displayed in Figure 4 and Supplementary Figure 2E-H. LFC as compared to respective non-ACLF group; Weighted LFC = LFC multiplied by -log(p).

[1]	Zhang Y, Tan W, Wang X, Zheng X, Huang Y, Li B, et al. Metabolic biomarkers significantly enhance the prediction of HBV-related ACLF occurrence and outcomes. J Hepatol 2023;79:1159-1171.
